# In Silico Analysis of Seven PCR Markers Developed from the *CHD1*, *NIPBL* and *SPIN* Genes Followed by Laboratory Testing Shows How to Reliably Determine the Sex of Musophagiformes Species

**DOI:** 10.3390/genes13050932

**Published:** 2022-05-23

**Authors:** Aleksandra Kroczak, Heliodor Wierzbicki, Adam Dawid Urantówka

**Affiliations:** Department of Genetics, Wrocław University of Environmental and Life Sciences, Kożuchowska 7, 51-631 Wrocław, Poland; aleksandra.kroczak@upwr.edu.pl (A.K.); heliodor.wierzbicki@upwr.edu.pl (H.W.)

**Keywords:** *CHD1* gene, molecular markers, Musophagiformes, *NIPBL* gene, sex determination, *SPIN* gene

## Abstract

Sex determination in birds, due to the very common lack of sexual dimorphism, is challenging. Therefore, molecular sexing is often the only reliable way to differentiate between the sexes. However, for many bird species, very few genetic markers are available to accurately, quickly, and cost-effectively type sex. Therefore, in our study, using 14 species belonging to the order Musophagiformes, we tested the usefulness of seven PCR markers (three of which have never been used to determine the sex of turacos), developed based on the *CHD1*, *NIPBL*, and *SPIN* genes, to validate existing and develop new strategies/methods of sex determination. After in silico analysis, for which we used the three turaco nuclear genomes available in GenBank, the suitability of the seven selected markers for sexing turacos was tested in the laboratory. It turned out that the best of the markers tested was the 17th intron in the *NIPBL* gene (not previously tested in turacos), allowing reliable sex determination in 13 of the 14 species tested. For the one species not sexed by this marker, the 9th intron in the *CHD1* gene proved to be effective. The remaining markers were of little (4 markers developed based on the *CHD1* gene) or no use (marker developed based on the *SPIN* gene).

## 1. Introduction

Modern birds (the subclass Neornithes), which are divided into two clades, Palaeognathae (ratites and flying tinamous) and Neognathae (all other extant birds), are represented by more than 10,000, highly diverse species, spread throughout the world. Such great diversity of extant birds was initiated by their ancestors that survived a mass extinction event (66 million years ago) and then evolved into such a large number of species [1]. Despite the high phenotypic variability that characterizes birds, sexual dimorphism is absent or hardly noticeable in many bird species (they are monomorphic). Even in sexually dimorphic birds, distinguishing sex in chicks based on morphological features is rarely possible. This causes serious problems because sex determination is crucial in ecological and demographic studies, in captive breeding for conservation programs, in efficient reproduction in zoos, and in scientific research [2,3,4]. Despite being challenging, there are many methods for sexing birds that can be divided into invasive (e.g., morphometric measurement, cloacal examination, laparotomy, and laparoscopy) and noninvasive (e.g., behavior observation, the ratio assay between estrogen and androgen in faces) [4,5,6]. However, the sexing methods listed are not accurate enough, and the invasive ones often put the birds at risk of injury. Therefore, molecular sexing of birds has become a non-invasive (or minimally invasive) and accurate method for reliable, rapid, and cost-effective sex differentiation.

The most widely used molecular methods to determine the sex of birds are based on PCR (Polymerase Chain Reaction) markers developed using the conserved *CHD1* (the Chromo Helicase DNA binding protein gene) [7,8,9,10], *NIPBL* (the Nipped-B homolog gene) [11], *SPIN* (the Spindlin gene) [12] and *RASA1* (the RAS p21 protein activator 1 gene) [13]. The basis of using these genes for avian sex typing is detecting differences between their copies located on Z and W sex chromosomes (in birds, a sex chromosomal system with ZW heterogametes in females and ZZ homogametes in males occurs [14,15]). One of these differences is the length polymorphism of introns in avian genes located on different sex chromosomes. This allows the amplification of specific gene fragments located on the Z and W sex chromosomes and their subsequent separation in agarose or polyacrylamide gels. In males, a single PCR product appears (a gene copies from the two Z chromosomes are usually of identical length), while in females, two products are detected due to the length polymorphism of the copies of genes located on the chromosomes Z and W [16]. Various methods and molecular markers used to determine the sex of many bird species have been described, applied or reviewed by a number of authors [17,18,19,20,21,22]. Despite attempts to find a single universal DNA marker useful for sexing birds, this has so far been unsuccessful [23].

**Figure 1:**
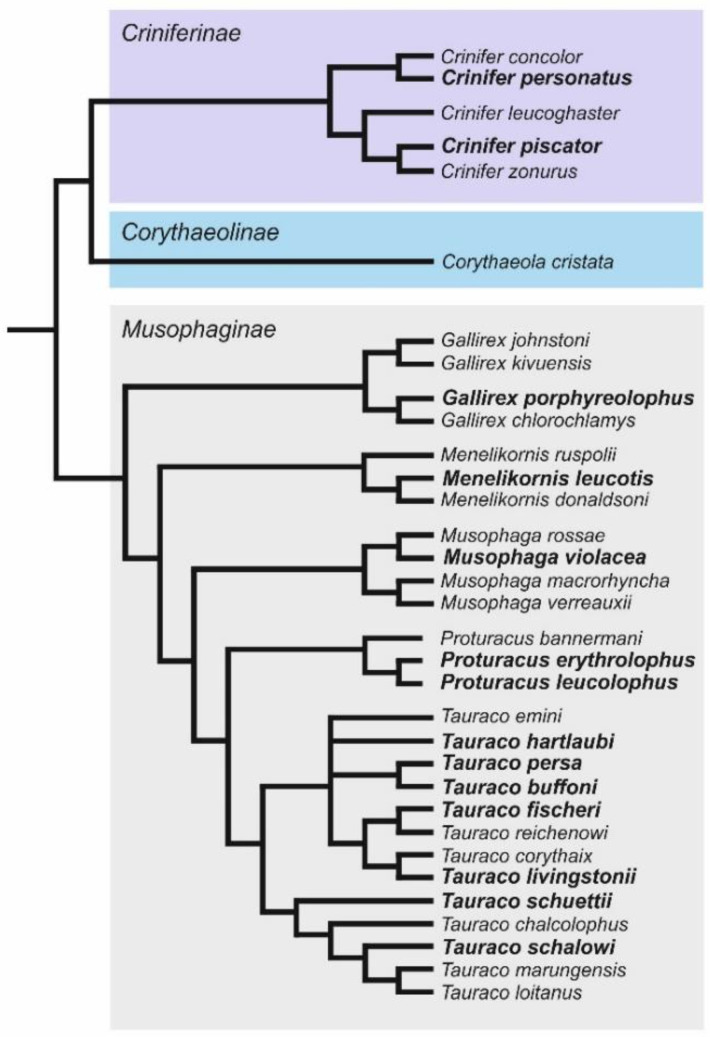
Phylogenetic tree showing genetic relationships among thirty-three species included in the order Musophagiformes [25]. The species studied in this paper are indicated in bold.

One of the many orders that belong to the class Aves are the turacos (Musophagiformes). They are endemic to Sub-Saharan Africa, arboreal, usually brightly colored, and almost exclusively herbivorous [24,25]. According to Perktaş et al. [25], who proposed a revised classification based on phylogeographic and phylogenetic analysis, thirty-three species in seven genera corresponding to the major clades (subfamilies: Criniferinae, Corythaeolinae, and Musophaginae) were recognized within the turacos (Figure 1).

With the exception of the white-bellied go-away-bird (*Crinifer leucogaster*), turaco species do not exhibit sexual dimorphism [26], therefore molecular sexing is the only reliable way to determine the sex of species in this avian order. To our knowledge, three PCR markers (primer sets) developed based on the *CHD1* gene were used for molecular sexing of the turacos: P2/P8 for sex typing of *Corythaeola cristata* [27], 1272H/1237L for sex typing of *C. cristata* [27] and *Tauraco persa* [8], and 2550F/2718R for sex typing of *T. persa* [8,19]. Additionally, markers developed using the *SPIN* gene (USP1/USP3 and INT-F/INT-R, multiplex PCR [28]) were used for sexing *Musophaga violacea* [29], P2/P8 + EcoRI (PCR-RFLP) for sexing *T. persa* [30], and P2/NP/MP (multiplex PCR) for sexing *T. persa* [31]. As can be seen from the studies cited, only three taxa of turacos have been molecularly sexed using these markers. In addition, some reports cannot be considered sufficiently reliable because images of the electrophoretic patterns of amplicons obtained for the individuals tested were not provided [8] or only one individual was studied [30,31]. Furthermore, when we used the Basic Local Alignment Search Tool (BLAST) to search the nuclear genomes of selected species of the order Musophagiformes, available in the database, we did not find the amplicon sequences used by Jensen et al. [27] for sex determination in *C. cristata*.

With this in mind, using a relatively large group of species representing the family Musophagidae (fourteen out of thirty-three recognized species), we tested the usefulness of seven PCR markers (three of which have never been used to determine the sex of turacos) to validate existing and develop new strategies/methods of sex determination for a wide range of turaco species. Here, we present the results of this investigation.

## 2. Materials and Methods

### 2.1. Biological Samples and DNA Extraction

Thanks to the courtesy of the Wrocław Zoological Garden and private breeding facilities in Poland, we were able to collect blood and feather samples from fourteen turaco species (86 individuals in total). From the Wroclaw Zoo, we received blood samples from male and female *M. violacea*, blood samples from male *Crinifer personatus*, and feather samples from female *C. personatus*. Blood samples of males and females of the remaining twelve turaco species came from private Polish breeding centers. The sex of the tested individuals was determined using a laparoscope (both *C. personatus* from the Wrocław Zoo), or blood samples were taken from selected breeding pairs that had repeatedly reared their offspring (the other species studied). Detailed information on the studied turaco species and differences in the names of the studied taxa suggested by different authors [25,32,33] are given in Table 1. In our study, we used the new classification of the family Musophagidae developed by Perktaş et al. [25].

Blood samples were collected for laboratory analysis as dry blood spots on a fiber filter to prevent potential microbial degradation of blood cells and degradation of genomic DNA. They were then preserved in parafilm-sealed Eppendorf tubes at −20 °C until use to avoid dampness. For freshly collected breast feather samples, the feather shafts were cut off and stored under the same conditions until use. Total DNA was extracted from both tissue types using the Sherlock AX Kit (A&A Biotechnology, Gdynia, Poland) according to the manufacturer’s protocol.

### 2.2. DNA Amplification

PCR amplifications were performed in 25 μL reaction mixture containing 50 ng of the DNA template, 1 U DreamTaq Green DNA Polymerase (Thermo Fisher Scientific, Waltham, MA, USA), 2.5 μL of 10 × buffer, 0.6 μL of 10 mM dNTPs, and 0.6 μL of each primer (10 μM).

We tested the use of seven different amplicons (named for the purpose of this study M1, M2, M3, M4, M5, M6 and M7) as potential markers for sexing turaco taxa using the previously described PCR primers. The details of the primers used (names, sequences and references) are given in Table 2.

The reaction conditions were as follows: the M1 program: 94 °C 5 min, (94 °C 30 s, 48 °C 30 s, 72 °C 60 s) 35 times, and 72 °C 5 min; the M2 program: 94 °C 5 min, (94 °C 30 s, 48 °C 30 s, 72 °C 60 s) 35 times, and 72 °C 5 min; the M3 program: 94 °C 5 min, (94 °C 30 s, 51 °C 30 s, 72 °C 90 s) 35 times, and 72 °C 5 min; the M4 program: 94 °C 5 min, (94 °C 30 s, 50 °C 30 s, 72 °C 60 s) 35 times, and 72 °C 5 min; the M5 program: 94 °C 5 min, (94 °C 30 s, 51 °C 30 s, 72 °C 60 s) 35 times, and 72 °C 5 min; the M6 program: 94 °C 5 min, (94 °C 30 s, 55 °C 30 s, 72 °C 60 s) 35 times, and 72 °C 5 min; the M7 program: 94 °C 5 min, (94 °C 30 s, 53 °C 30 s, 72 °C 60 s) 35 times, and 72 °C 5 min. For each amplified fragment, 5 µL of the PCR reaction mixtures was loaded onto the 1%, 2% or 3% agarose gels. The gel contained ethidium bromide at a concentration of 0.5 µg/mL. The electrophoresis was performed in 1× TAE buffer at a voltage of 5 V/cm.

### 2.3. In Silico Analysis of the Genetic Markers Tested

Genome resources from the GenBank database were searched for nuclear genomes of Musophagiformes. The available genomes were checked for annotation of the chromosomes and genes located on them. The genome of male *T. erythrolophus* (GenBank assembly accession: GCA_000709365.1) was used to analyze the structure of the *CHD1*, *SPIN,* and *NIPBL* genes. Their entire sequences and exon sequences were aligned using the MEGA program [36] to determine the length and position of the introns within the genes analyzed. Next, to predict the size of the potential Z-specific amplicons for the seven markers analyzed, the appropriate forward and reverse primers were aligned with the sequences of the *CHD1*, *SPIN*, and *NIPBL* genes using the MEGA program [36]. To define the position of characterized Z-specific amplicons within the genes analyzed, the appropriate primer locations were compared with their exon/intron structure. To find W-specific amplicons, the genome of female *T. erythrolophus* (GenBank assembly accession: GCA_009769465.1) was searched using BLAST (Basic Local Alignment Search Tool) and the sequences of identified Z-specific amplicons as queries. The two-copy sequences (Z- and W-specific) of the markers tested were aligned using the MUSCLE algorithm [37] in MEGA [36]. The same approach was used for the rest of the available turaco genomes.

### 2.4. In Silico Analysis of the Gene Content of T. erythrolophus Chromosomes

Genome Data Viewer [38] was used to check if genes identified by Smeds et al. [39] on the Z chromosome of *Collared flycatcher* (*Ficedula albicollis*) are also located on the Z chromosome of *Melopsittacus undulatus* (NC_047557). The exon sequences for 42 identified genes were then downloaded. Using BLAST and selected exons of these genes as query sequences, we searched the nuclear genome of female *T. erythrolophus*. Information on their genomic location was used to determine their chromosomal positions.

## 3. Results and Discussion

### 3.1. In Silico Analysis of the Genetic Markers Tested

Before laboratory testing, in silico analysis of seven selected PCR markers (M1, M2, M3, M4, M5, M6, M7), which are introns located in three genes (*CHD1*, *SPIN,* and *NIPBL*) was performed. Its purpose was to verify whether the amplicon sequences to be used for turaco sexing have Z and W-specific copies (variants located on the Z and W sex chromosomes) and whether there is a polymorphism in their length that would allow distinguishing male from female.

Searching GenBank resources, we found nuclear genomes of three species of turacos: *Crinifer concolor* (GenBank assembly accession: GCA_013399495.1-BioSample: SAMN12253899, female only); *C. cristata* (GenBank assembly accession: GCA_013396815.1-BioSample: SAMN12253763, male only), and *T. erythrolophus* (GenBank assembly accession: GCA_000709365.1-BioSample: SAMN02339893—for male, and GenBank assembly accession: GCA_009769465.1-BioSample: SAMN12621036—for female). After analysis of the retrieved nuclear genomes, it turned out that only scaffolds with unknown gene coding regions (CDSs-coding DNA sequences) were available for the first two species. Furthermore, there was no information on their location on the avian chromosomes. For the third species (*T. erythrolophus*), 16,054 CDS were identified in the male genome (but the number and length of chromosomes has not been determined), while no CDSs were provided in the female genome, but 33 chromosomes were identified in its assembly (31 +Z/W). Since CDSs were only determined in the genome of male *T. erythrolophus*, this genome was used to analyze the structure of genes containing marker sequences (*CHD1*, *SPIN,* and *NIPBL*).

For the *CHD1* gene (GenBank assembly accession NW_010038079.1—73,805 bp) and the *SPIN* gene (GenBank assembly accession NW_010072932.1—49,958 bp), their entire sequences and exon sequences could be found (exon sequences: *CHD1*-XM_009991467.1—mRNA length 6.776 bp; *SPIN*-XM_009988563.1—mRNA length 4.346 bp). However, intron sequences were not available. Regarding the *NIPBL* gene, four sequences were available for four different loci (LOC104371354, LOC104370058, LOC104383964 and LOC104377353). To select the right one for our study, we used BLAST (Basic Local Alignment Search Tool) to search the nuclear genome of *Tauraco erythrolphus*. Using the Z-specific sequence of intron sixteen in the *NIPBL* gene (NIPBLi16) of *Columba palumbus* (Accession: JF279580.1) as a query, we searched the Z chromosome (WOXW01000129.1-93,020,717 bp) of female *T. erythrolphus* (assembly GCA_009769465.1). As a result, we found the corresponding sequence of length 801 bp, which was located at LOC104377353. Therefore, we found that the sequence of this locus (out of four available in GenBank) is the correct *NIBPL* gene for our analysis (GenBank assembly accession NW_010032872.1—102.535 bp; exon sequences-XM_009984506.1—mRNA length 4.972 bp).

The selected sequences allowed us to determine the exact position and length of exons and introns (Supplementary Table S1) within the *CHD1*, *SPIN*, and *NIPBL* genes. We then related the position of the primers used to amplify markers M1–M7 (Table 2) to the positioned exons and introns. In this way, we determined the sizes and sequences of potential Z-specific amplicons (Table 3), as well as which introns would be amplified and the lengths of the exons flanking them (Table 3). We found that M2 is an inner variant of M1, and M5 is an inner variant of M4 (it means that the same intron is amplified by different primers located on different positions in the flanking exons; this is indicated by the mutual location of the following primers: P8 and 1272 H; P2 and 1237 L; 2550 F and CHD1i16-F; 2718R and CHD1i16-R). Our analysis also showed that the *NIPBL* primers in *T. erythrolphus* amplify intron 17 rather than intron 16. Unfortunately, we could not obtain information for *SPIN* because the MEGA program did not find sequences within this gene that matched the sequences of the primers used for M6 (Table 3).

Afterwards, Z-specific M1–M5 and M7 amplicons obtained for male *T. erythrolophus* (BioSample: SAMN02339893), were used to search the genome of the female of this species (BioSample: SAMN12621036). This analysis revealed that the amplicon sequences for M1–M5 and M7 identical in length and sequence (100% identity) to those previously found in the male were located on the Z chromosome in the female (GenBank WOXW01000129.1—93,020,717 bp) (Table 4). Interestingly, second copies of the sequences of these amplicons were additionally found in the female genome, but differed in length from the Z-specific copies. For M1 and M2, the length difference was 11 bp, for M3 it was 2374 bp, for M4 and M5 it was 43 bp, and for M7 it was 413 bp. The Z-specific copy was shorter for M1 and M2, while for M3, M4, M5 and M7 the Z-specific copy was longer. This indicates the possible use of these markers for sex typing (length polymorphism occurs). The results of this alignment are visualized in Supplementary Figure S1.

The same in silico analysis as for the female *T. erythrolophus* was conducted for female *C. concolor* (BioSample: SAMN12253899) and male *C. cristata* (BioSample: SAMN12253763). In female *C. concolor*, two copies of M1, M2, M4, and M5 were found on different scaffolds (VXAM01002689.1—14,797 bp, and VXAM01001990.1—45,938 bp). However, for these four markers, both copies were identical in length. For M3 and M7 only one copy was found. The length of the M3 sequence corresponds to the longer copy, and the length of the M7 sequence corresponds to the shorter copy previously found in *T. erythrolophus* (Table 4). No M1 and M2 amplicon sequences were found in the genome of male *C. cristata*, and only one sequence corresponding to the Z-specific copy of male and female *T. erythrolophus* was correctly found for M3. Additionally, for M4 and M5 only one sequence was found for each amplicon. Both amplicon sequences were much longer than the sequences found for *C. concolor* and *T. erythrolophus* (Table 4). In the case of M7, only a fragment of the amplicon sequence was found at positions 1 to 525 of the 1255 bp long WBMX01031976.1 scaffold, corresponding to positions 398 to 923 of the reference sequence. So, it seems that this is not a shorter version of the amplicon, but an incomplete longer version.

Only in the case of intron 17 in the *NIPBL* gene (M7) its second copy was found on the W chromosome (WOXW01000067.1—29,633,611 bp). The other second copies of the markers tested (M1–M5) were located on scaffold 37 (GenBank: WOXW01000076.1) with a length of 1,556,041 bp. Therefore, the question arises of whether the second copy of the *CHD1* gene is located on the W chromosome. To answer this question, exons and introns of the *CHD1* gene (Supplementary Table S1) were used as reference sequences (queries) to BLAST the genome of female *T. erythrolophus*. The results of this analysis are shown in Supplementary Table S2. Unfortunately, for most queries, analogous sequences were found on scaffold 37, where the query cover for exons and introns was 92–100% (identity 83.45% to 97.62%) and 10–100% (identity 66.78% to 92.32%), respectively. Only one sequence was found on the W chromosome that strongly matches the Z-copy—the 9th intron with a query cover of 80% and an identity of 84.30%. The other sequence found—the 1st intron—had a higher identity (92.89%), but with a very low query cover of 16%. On the contrary, all identified exons of the *NIPBL* gene (Supplementary Table S1) have their counterparts on the W chromosome with a query cover of 99–100% (with identities ranging from 90.42% to 98.89%), and most introns (except intron 11) have their copies on the W chromosome where the query cover ranges from 40% to 100% with identities ranging from 74.72% to 99.41% (Supplementary Table S3). In light of these results, it is difficult to determine with certainty whether scaffold 37 is a fragment of an incorrectly assembled W chromosome or whether the W copy of the *CHD1* gene has translocated to another part of the nuclear genome.

**Figure 2:**
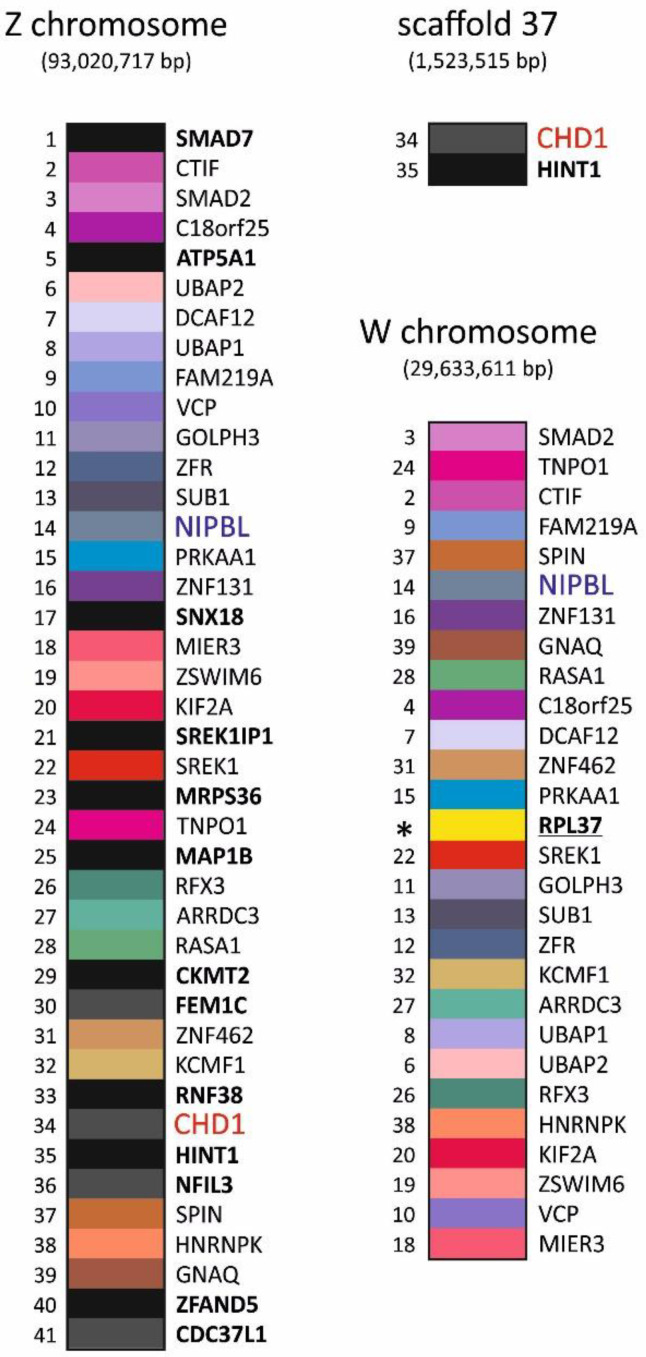
Gene content of the red-crested turaco (*T. erythrolophus*) Z chromosome, W chromosome and nuclear scaffold 37. The names of genes identified on the Z chromosome that do not have their counterparts on the W chromosome are marked in bold. The name of a gene identified on the W chromosome that has no counterpart on the Z chromosome is underlined. Its position on the W chromosome is marked with an asterisk (*). On the W chromosome and Scaffold 37, the gene numbering was retained to correspond to the position of their counterparts on the Z chromosome.

In an attempt to answer this question we determined the gene contents of the *T. erythrolophus* Z chromosome (WOXW01000129.1—93,020,717 bp), W chromosome (WOXW01000067.1—29,633,611 bp) and scaffold 37 (WOXW01000076.1—1,556,041 bp). We searched for 42 genes identified by Smeds et al. [39] on the Z chromosome in *C. flycatcher* (*F. albicollis*) (see Table 1 in the cited paper; two genes were omitted in our analysis: *novel1* and *novel2*). This analysis revealed that 41 of the 42 selected genes were found on the Z chromosome of *T. erythrolophus*. Information on their chromosomal location allowed us to determine their positions on the Z chromosome (Figure 2). Only one of these 42 genes (*RPL37*) was found on the W chromosome. The 14 genes located on the Z chromosome (indicated in bold and *CHD1* indicated in red) were not found on the W chromosome. The *CHD1* gene and *HINT1* were identified on scaffold 37. Therefore, it is still unclear what scaffold 37 is, whether it is a microchromosome or a missing fragment of an incorrectly assembled W chromosome.

To summarize our in silico analysis, we concluded that there is a need for laboratory verification of the presence of the sequences of the markers studied (M1–M7) in the nuclear genomes of the fourteen turaco species analyzed and to identify possible length differences between the Z-copies and the second copies of these amplicons. Except for *NIPBL* (M7) having a second copy located on the W chromosome, it is not known whether the second copies of markers associated with the *CHD1* gene are located on the W chromosome or in another part of the genome (this question requires further research). In this context, it is of particular interest whether markers M1–M5 can still be considered sex-specific and whether there are more than two copies of these amplicons due to their potential jumping in the genome (perhaps they are TEs -Transposable Elements). This scenario cannot be ruled out because according to Peona et al. [40], the density of transposable elements of the avian W chromosome exceeds 55%, compared to less than 10% genome-wide density. An earlier study by Suh et al. [11] also detected insertions of retroposons (mobile elements that integrate randomly in genomes) into introns 9 and 16 of the avian *CHD1* gene.

### 3.2. Laboratory Verification of the Usefulness of the Tested Markers for Sex Typing in the Turaco Species Studied

Laboratory tests of PCR markers developed based on the *CHD1* gene (M1–M5), the *SPIN* gene (M6), and the *NIPBL* gene (M7) allowed us to verify information (earlier obtained through our in silico analysis and previously published sparse reports [8,19,27]) on their suitability for sex typing of 14 studied species belonging to the order Musophagiformes, and to recommend those PCR markers that reliably, rapidly, and cost-effectively enable sex determination in a wide range of species. It is worth emphasizing that three of the seven markers we tested (M3, M5, and M7) have never been used to determine the sex of turacos.

#### 3.2.1. Polymorphism of the Studied Introns in the CHD1 Gene

**Figure 3:**
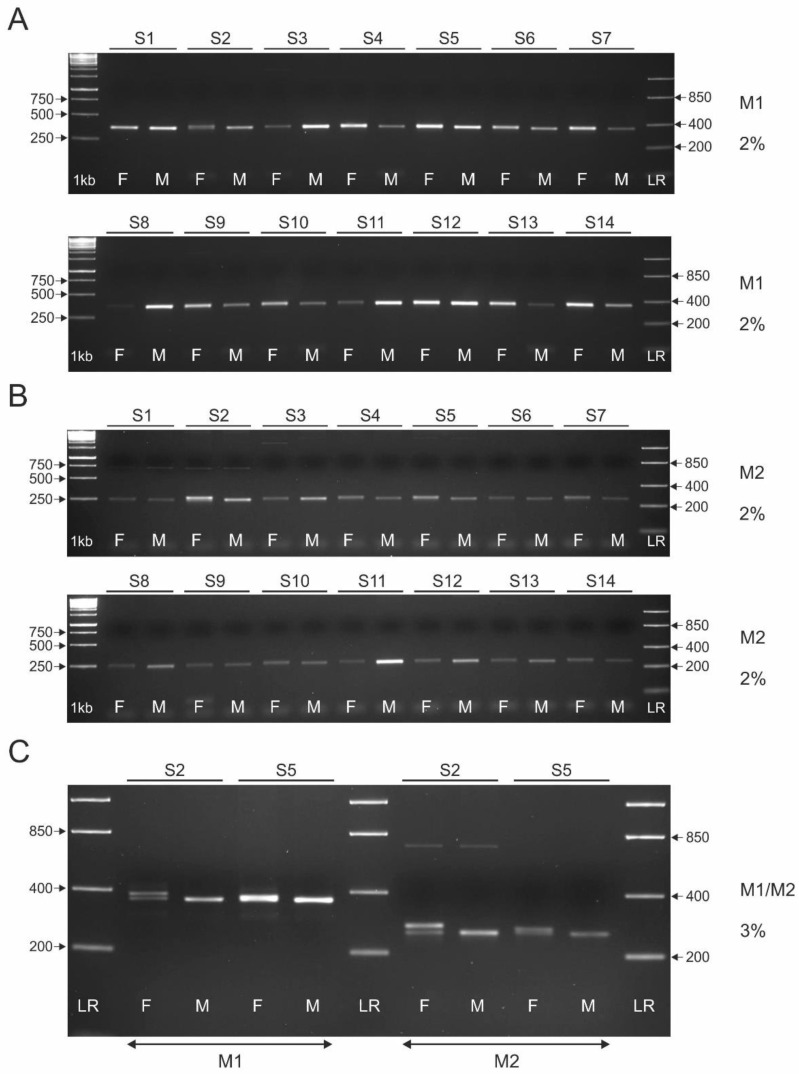
Patterns of the PCR products representing M1 (**A**) and M2 (**B**) markers obtained for male (M) and female (F) individuals of fourteen turaco species (S1–S14, see Table 1). The amplicons were resolved in 2% agarose gel. The amplicons resolved in 3% agarose gel (S2 and S5) are shown in (**C**). Lane 1 kb—GeneRuler 1 kb DNA Ladder (Thermo Scientific, Waltham, MA, USA). Lane LR—FastRuler Low Range DNA Ladder (Thermo Scientific).

**Figure 4:**
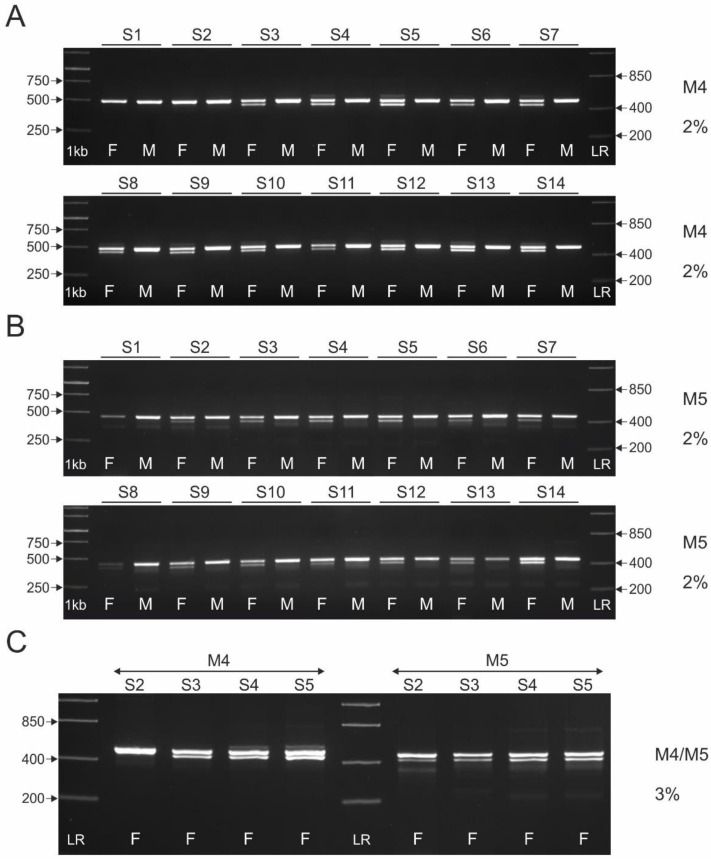
Patterns of the PCR products representing M4 (**A**) and M5 (**B**) markers obtained for male (M) and female (F) individuals of fourteen turaco species (S1–S14, see Table 1). The amplicons were resolved in 2% agarose gel. The female amplicons resolved in 3% agarose gel (S2–S5) are shown in (**C**). Lane 1 kb—GeneRuler 1 kb DNA Ladder (Thermo Scientific). Lane LR—FastRuler Low Range DNA Ladder (Thermo Scientific).

**Figure 5:**
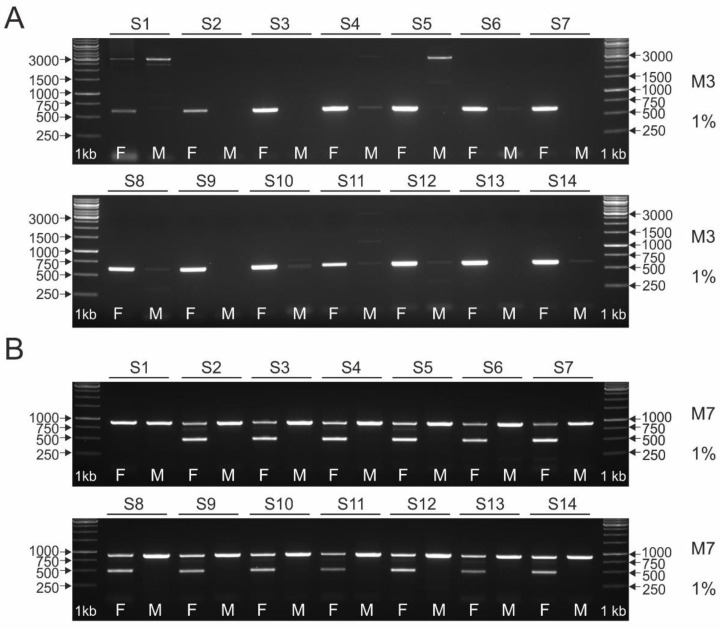
Patterns of the PCR products representing M3 (**A**) and M7 (**B**) markers obtained for male (M) and female (F) individuals of fourteen turaco species (S1–S14, see Table 1). The amplicons were resolved in 1% agarose gel. Lane 1 kb—GeneRuler 1 kb DNA Ladder (Thermo Scientific).

The results of using intron 22 (markers M1 and M2), intron 16 (markers M4 and M5), and intron 9 (marker M3) in the *CHD1* gene as molecular markers for sex determination of fourteen turaco species (S1–S14, see Table 1) are shown in Figure 3, Figure 4 and Figure 5A, respectively.

We used a 2% agarose gel to test M1 and M2 (Figure 3A,B) because our in silico analysis for *T. erythrolophus* indicated that a small size difference (11 bp) between the Z and W-specific copies of these markers would be expected (Table 4). In the case of M1 (Figure 3A), only one Z-specific amplicon of ~380 bp in length was detected for all species tested, which corresponds to our earlier performed in silico analyzes. The W-specific amplicon is not visible, which may be due to its length being similar to that of the Z-specific amplicon (they cannot be separated in a 2% agarose gel). However, the indistinct band for the female S2 may indicate the presence of two unseparated fragments. A similar situation was observed for M2, which is an inner variant of M1 (Figure 3B). Only one Z-specific amplicon with a length of 250–260 bp is visible in all tested species, which corresponds to the results of in silico studies. If the W-specific amplicon is present, it is very similar in size to the Z-specific amplicon, and in a 2% agarose gel the two fragments cannot be separated. However, the indistinct band for S2 female may indicate the presence of two unseparated fragments. Furthermore, nonspecific bands of ~700 bp are observed in S1 and S2, and additional nonspecific bands of ~2000 bp and ~4000 bp are observed in S3 and S5, respectively. As potentially unseparated bands were observed in the 2% agarose gel for S2, we used a 3% agarose gel to separate the M1 and M2 amplicons obtained for both S2 and S5 (Figure 3C). For S2, we were able to separate both M1 and M2. However, a very small length difference was found that corresponded to ~10 bp. Furthermore, for S5 it was possible to separate M2, but the difference in length of the fragments was very small and it was difficult to visually distinguish the two bands.

Markers M1 and M2 were previously tested for sexing two species belonging to the order Musophagiformes: *C. cristata* [27] and *T. persa* [8]. For *C. cristata* (we did not study this species in the laboratory), the authors reported that both M1 and M2 can be used for sex determination. However, in our in silico study (Table 4), when we searched the nuclear genome of this species (using BLAST), we could not find the sequences of both amplicons. In the case of *T. persa*, the cited authors tested M2, showing that this marker effectively determines sex. However, the authors did not provide images of the banding patterns or the number of males and females tested. In our study, M2 did not allow sex typing in *T. persa* due to the small difference in length between Z and W-specific amplicons. In conclusion, M1 and M2 are not useful for sexing the studied turaco species using standard percentages of agarose gels. It is due to very small size differences between Z and W-specific amplicons, which are impossible to visualize in 1% and 2% agarose gel (Figure 3A,B). The use of a 3% agarose gel allowed for separation Z and W-specific amplicons only in the case of S2 (M1 and M2) and S5 (M2) (illustrated in Figure 3C). It cannot be ruled out that M1 and M2 might be suitable for sexing turacos if high resolution equipment is used (polyacrylamine gels or capillary sequencers). However, these methods are more expensive and more elaborate, and thus less easy to use.

To test the usefulness of M4 and M5 in typing the sex of turacos, as with M1 and M2, we used a 2% agarose gel because in silico analysis (Table 4) indicated that a small size difference (43 bp) between Z and W-specific amplicons should be expected. In the case of M4, two separated Z and W-specific bands are seen for twelve (S3–S14) of the fourteen species studied (Figure 4A). The Z-specific band of 490–500 bp matches the predictions of the in silico analysis (504 bp for *T. erythrolophus*). The W-specific amplicon of 460–470 bp in length is also clearly visible. In the case of S1 and S2, this marker proved to be useless as only the Z-specific amplicon is visible. For most of the remaining (successfully sexed) species, a faintly visible additional band above the Z-specific amplicon is visible in females. One might speculate why these additional faintly visible bands were detected. If we assume that the Z-specific M4 was duplicated, jumped elsewhere in the genome, and then some mutations occurred in the primer annealing sites of the copied sequence, this could have altered the primer annealing ability. As a result, additional PCR products (of varying lengths) may have appeared as faintly visible bands. This also raises the question of the location of the second copy of the *CHD1* gene in the genome, is it located on the W chromosome, and if so, is it the only copy of the *CHD1* gene (our in silico study did not find the *CHD1* gene on the W chromosome, but located it in scaffold 37). These questions remain unanswered for now.

The M5 marker (which is an inner variant of M4) differentiates sex in the thirteen species studied, only in the case of S1 it is useless (Figure 4B). The Z-specific amplicon of 460–470 bp, corresponding to our prediction from in silico analysis, is separated from the W-specific amplicon of 420–430 bp. For many species studied (both males and females), nonspecific bands of ~380 bp in length are visible.

As the 2% agarose gel in S2 showed two separated bands for the M5 marker, but in the same individual two bands could not be detected for M4, samples of selected females (S2–S5) were tested on a 3% agarose gel to make sure how many amplicons (one or two) for these markers are actually produced (Figure 4C). The results of this test revealed the unsuitability of the M4 marker for S2 sexing even in 3% agarose gel, and confirmed the small difference in the size of Z and W-specific amplicons in the other species tested. In conclusion, M4 and M5, due to the small difference in the length of Z and W-specific amplicons, are not reliable and easily interpretable markers when used to determine the sex of turacos, especially since additional bands are present in female samples for M4, and some nonspecific bands for M5 are observed in samples of both sexes.

The suitability of the M4 marker for sexing turacos (*T. persa* only) was studied by Vuicevic et al. [19] and Wang et al. [8]. Both authors reported the inability to determine the sex of the species studied using this marker (the first authors tested only two males, while the second authors reported only the conclusion without providing images of banding patterns). In our study, in *T. persa*, both Z and W-specific bands were separated and visualized in a 2% agarose gel. Regarding the M5 marker, to our knowledge it has not been previously tested for sex determination in turacos.

The M3 marker (CHD1i9) due to the distinct differences in the size of the Z and W amplicons (500 bp) was suggested for molecular sex determination of Neoaves [11]. However, so far it has not been tested in species belonging to the order Musophagiformes. The results of our analysis showing the suitability of this marker for sexing turacos are shown in Figure 5A. A single amplicon of ~600 bp is evident for females of all species, which corresponds with the results of our in silico analysis (Table 4). Unfortunately, a faint band, ~600 bp in length, appears in many males. This is not necessarily the same band as in females, but it is difficult to determine this unambiguously in a 1% agarose gel (a 1% agarose gel was used because the expected difference in length between Z and W-specific amplicons was 2374 bp; see Table 4). The predicted in silico Z-specific amplicon of ~3000 bp length appears only in S1, S5 and S11 males, as well as in S1 female. In addition, a third additional band of ~1300 bp in length appears in S2 and S11 males. Due to the banding pattern, the M3 marker is of little use for sexing the vast majority of species tested (S2–S14). It is specific mainly to W amplicons, and additional bands make interpretation of the result difficult. The only species tested for which M3 can be used for sex determination is S1, because in the female it yields in silico predicted amplicons of 600 bp and 3000 bp. The additional 2400 bp amplicon observed in the male S1 does not affect the interpretation, because in this species no artifact appears in the male (a nonspecific band at a level that corresponds to the female 600 bp band).

Reports by other authors on the use of PCR markers for sex determination in turacos describe the use of more time-consuming and expensive methods such as PCR-RFLP (primer set P2/P8 + EcoRI) [30] and multiplex-PCR (primer set P2/NP/MP) [31]. In both cases, the authors tested *T. persa*. Although the reported results confirm the efficacy of the methods and markers tested (developed from the *CHD* gene), their reliability should be treated with caution. In the first case [30], only one female sample was tested and the authors did not provide images of banding patterns, and in the second case [31] only one sample, supposedly a male, was tested.

#### 3.2.2. Length Polymorphism of the 17th Intron in the NIPBL Gene

The M7 marker (NIPBLi17), like M3, has not been previously tested for sex typing in turaco species. It was recommended by Suh et al. [11] as a reliable marker for sex determination of Neoaves. It appears in the literature as NIPBLi16 (intron 16 of the *NIPBL* gene). However, our thorough in silico analysis revealed that M7 in the order Musophagiformes is the 17th intron in the *NIPBL* gene (see Table 3). The results of testing this marker for sex typing in turacos are shown in Figure 5B. The M7 marker proved to be reliable and easy to interpret in determining the sex of the 13 species studied (S2–S14). Two bands, a ~900 bp long Z-specific, and a ~500 bp long W-specific, are clearly visible in a 1% agarose gel. The lengths of the Z and W amplicons detected correspond to our in silico predictions. For S1, only the Z-specific amplicon was detected, for both the female and the male, indicating that this marker is not useful for this species.

#### 3.2.3. Testing of the M6 Marker Developed on the Basis of the SPIN Gene

Although our in silico analysis did not reveal Z and W-specific amplicon sequences representing the M6 marker in the turaco genomes examined (Table 4), we decided to test its utility by using the primer set USP1/USP3, which allows amplification of a W-chromosome specific amplicon. However, the use of this marker may not give reliable and conclusive results because the absence of an amplicon may indicate that the sample tested is a male, or it may falsely indicate a male due to degraded DNA (false identification of a male may then result from a mismatch between primers and a potentially recognized annealing site in the species tested). The results of our analysis are shown in Supplementary Figure S2. A W-specific amplicon of ~390 bp is clearly visible in females of species S2, S4 and S5. However, no Z-specific amplicons were detected in these species (both males and females). A faintly visible W-specific amplicon was also detected in female S3. No amplicons were detected in the remaining species (S1, S6–S14), which would indicate that there is a mismatch between the sequence at the *SPIN* gene annealing site and the primer sequence. Thus, it appears, that the M6 marker is not useful for sex determination of the species tested.

Markers developed from the *SPIN* gene (primer sets USP1/USP3 and INT-F/INT-R) for sex typing of a turaco species, *M. violacea*, were tested by Itoh et al. [29]. The authors used multiplex-PCR and reported that both markers successfully sexed the species tested. However, when standard PCR is used, the set of primers USP1/USP3 produces only a W-specific amplicon.

**Figure 6:**
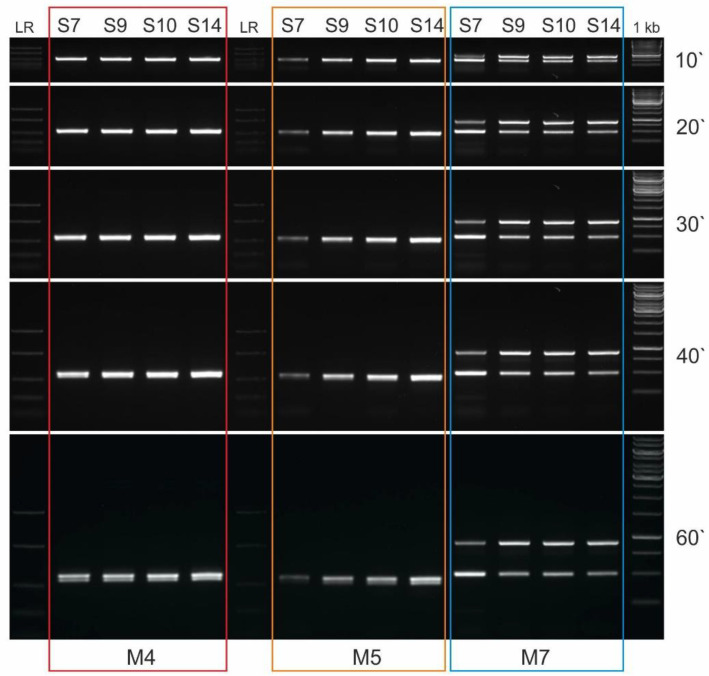
Patterns of the PCR products representing M4, M5 and M7 markers obtained for female individual of four turaco species (S7, S9, S10, and S14, see Table 1). The image shows the separation of amplicons in a 1% agarose gel after 10, 20, 30, 40 and 60 min of electrophoresis. Lane 1 kb—GeneRuler 1 kb DNA Ladder (Thermo Scientific). Lane LR—FastRuler Low Range DNA Ladder (Thermo Scientific).

To summarize our research, the PCR marker for sex determination of species belonging to the order Musophagiformes giving the most reliable and easy to interpret results is M7 (intron 17 in the *NIPBL* gene), which allows sex typing in thirteen (S2–S14) of the species we studied. The M3 marker (intron 9 in the *CHD1* gene) proved to be the only marker allowing sex determination in S1. Although both M4 and M5 can be used for many turaco species (to determine the sex of S3–S14, and S2–S14, respectively), the M7 marker is more useful because its PCR products can be successfully separated in a 1% agarose gel. An additional advantage of this marker is that the sexing results (size difference between Z and W specific amplicons) can be visualized after only 10 min of electrophoresis (Figure 6). For M4 and M5, two amplicons are detected in females after 60 min of electrophoresis.

## Figures and Tables

**Table 1:** Turaco species used in the reported research.

Species			Source	Designation	Male	Female
Clements et al. [32]	Gill et al. [33]	Perktaş et al. [25]				
***Corythaixoides personatus***	***Crinifer personatus***	***Crinifer personatus***	Wrocław Zoo	S1	1	1 *
*Crinifer piscator*	*Crinifer piscator*	*Crinifer piscator*	captive, Poland	S2	1	1
*Gallirex porphyreolophus*	*Gallirex porphyreolophus*	*Gallirex porphyreolophus*	captive, Poland	S3	1	6
***Tauraco leucotis***	***Menelikornis leucotis***	***Menelikornis leucotis***	captive, Poland	S4	1	2
*Musophaga violacea*	*Musophaga violacea*	*Musophaga violacea*	Wrocław Zoo	S5	3	4
***Tauraco erythrolophus***	***Tauraco erythrolophus***	***Proturacus erythrolophus***	captive, Poland	S6	19	13
***Tauraco leucolophus***	***Tauraco leucolophus***	***Proturacus leucolophus***	captive, Poland	S7	1	2
***Tauraco persa bufoni***	***Tauraco persa bufoni***	***Tauraco buffoni***	captive, Poland	S8	1	3
*Tauraco fischeri*	*Tauraco fischeri*	*Tauraco fischeri*	captive, Poland	S9	1	1
*Tauraco hartlaubi*	*Tauraco hartlaubi*	*Tauraco hartlaubi*	captive, Poland	S10	5	2
*Tauraco livingstonii*	*Tauraco livingstonii*	*Tauraco livingstonii*	captive, Poland	S11	1	3
*Tauraco persa persa*	*Tauraco persa persa*	*Tauraco persa*	captive, Poland	S12	3	5
*Tauraco schalowi*	*Tauraco schalowi*	*Tauraco schalowi*	captive, Poland	S13	2	1
*Tauraco schuettii*	*Tauraco schuettii*	*Tauraco schuettii*	captive, Poland	S14	1 *	1

Differences in the names of the tested taxa suggested by different authors are indicated in bold. Asterisks (*) indicate feather samples, others are blood samples.

**Table 2:** PCR primers used in this study.

Marker	Primer		
	Name	Sequence	Reference
**M1**	P2	TCTGCATCGCTAAATCCTTT	[34]
	P8	CTCCCAAGGATGAGRAAYTG	[34]
**M2**	1272H	TCCAGAATATCTTCTGCTCC	[35]
	1237L	GAGAAACTGTGCAAAACAG	[35]
**M3**	CHD1i9-F	CAGCAGAAATCAATCCAAGAC	[11]
	CHD1i9-R	CAGCCCATTTAACTGATAATCTC	[11]
**M4**	2550F	GTTACTGATTCGTCTACGAGA	[17]
	2718R	ATTGAAATGATCCAGTGCTTG	[17]
**M5**	CHD1i16-F	GTCCTGATTTTCTCACAGATGG	[11]
	CHD1i16-R	ATGATCCAGTGCTTGTTTCC	[11]
**M6**	USP1	CTATGCCTACCACMTTCCTATTTGC	[28]
	USP3	AGAAGATGSWCTGAARTCCAGCT	[28]
**M7**	NIPBLi16-F	TTGTCAGAGTTGCTGGAGATAC	[11]
	NIPBLi16-R	AATTTGATGGCACATAACTGTAG	[11]

**Table 3:** Components of M1–M7 amplicons predicted based on the location of the appropriate primer pairs within the *CHD1*, *SPIN*, and *NIPBL* genes. The lengths of the amplified exon (E) and intron (I) fragments are given in brackets.

Marker	Primer		Amplicon Length (bp)	Gene—Localisation
	Name	Position		
**M1**	P2	43,533	367	*CHD1*-E22(30 bp)/I22 (185 bp)/E23(152 bp)
	P8	43,167		
**M2**	1272H	43,178	258	*CHD1*-E22(19 bp)/I22 (185 bp)/E23(54 bp)
	1237L	43,435		
**M3**	CHD1i9-F	29,350	2997	*CHD1*-E9 (43 bp)/I9 (2852 bp)/E10 (102 bp)
	CHD1i9-R	32,346		
**M4**	2550F	38,324	504	*CHD1*-E16 (112 bp)/I16 (335 bp)/E17 (57 bp)
	2718R	38,827		
**M5**	CHD1i16-F	38,358	464	*CHD1*-E16 (78 bp)/I16 (335 bp)/E17 (51 bp)
	CHD1i16-R	38,821		
**M6**	USP1	unknown	unknown	*SPIN*-unknown
	USP3	unknown		
**M7**	NIPBLi16-F	68,920	923	*NIPBL*-E17 (54 bp)/I17 (801 bp)/E18 (68 bp)
	NIPBLi16-R	69,842		

**Table 4:** Turaco species analyzed in this study for length polymorphisms of the ninth, sixteenth, and twenty-second intron of the *CHD1* gene and the seventeenth intron of the *NIPBL* gene. The accession numbers and lengths of the identified fragments, as well as their assigned sex and biosample numbers, are shown. A single (*) or double (**) asterisk indicates different copies of the respective introns identified for *T. erythrolophus*. A copy with only a partial sequence identified for *C. cristata* is indicated with (p).

Species	Sex	Biosample	M1		M2		M3	
			Accession	L (bp)	Accession	L (bp)	Accession	L (bp)
*C. concolor*	Female	SAMN12253899	VXAM01002689.1	364	VXAM01002689.1	255	VXAM01004202.1	2996
*C. concolor*	Female	SAMN12253899	VXAM01001990.1	364	VXAM01001990.1	255	−	−
*C. cristata*	Male	SAMN12253763	−	−	−	−	WBMX01005567.1	2975
*T. erythrolophus*	Male	SAMN02339893	NW_010038079.1	367	NW_010038079.1	258	NW_010038079.1	2997
*T. erythrolophus*	Female	SAMN12621036	WOXW01000129.1	367 *	WOXW01000129.1	258 *	WOXW01000129.1	2997 *
*T. erythrolophus*	Female	SAMN12621036	WOXW01000076.1	378 **	WOXW01000076.1	269 **	WOXW01000076.1	623 **
**Species**	**Sex**	**Biosample**	**M4**		**M5**		**M7**	
			**Accession**	**L (bp)**	**Accession**	**L (bp)**	**Accession**	**L (bp)**
*C. concolor*	Female	SAMN12253899	VXAM01003074.1	503	VXAM01003074.1	463	−	−
*C. concolor*	Female	SAMN12253900	VXAM01001990.1	503	VXAM01001990.1	463	VXAM01001797.1	510
*C. cristata*	Male	SAMN12253763	WBMX01003140.1	672	WBMX01003140.1	632	WBMX01031976.1^(p)^	526 ^(p)^
*T. erythrolophus*	Male	SAMN02339893	NW_010038079.1	504	NW_010038079.1	464	NW_010032872.1	923
*T. erythrolophus*	Female	SAMN12621036	WOXW01000129.1	504 *	WOXW01000129.1	464 *	WOXW01000129.1	923 *
*T. erythrolophus*	Female	SAMN12621036	WOXW01000076.1	461 **	WOXW01000076.1	421 **	WOXW01000067.1	510 **

## Data Availability

Our research does not involve any nucleotide or amino acid sequence data which should be archived in publicly accessible repositories. All PCR patterns obtained are presented in figures included in the manuscript.

## Supplementary Materials

The following supporting information can be downloaded at: https://www.mdpi.com/article/10.3390/genes13050932/s1, Table S1: Number, position and length of exons and introns identified in CHD1, SPIN and NIPBL genes; Table S2: Identity of sequences homologous to the exons and introns of the *Tauraco erythrolophus* CHD1-Z gene, that were identified within Scaffold 37 or the W chromosome of this species; Table S3: Identity of sequences homologous to the exons and introns of the *Tauraco erythrolophus* NIPBL-Z gene, that were identified within the W chromosome of this species; Figure S1: Two-copy (Z and X-specific) sequence comparison of six markers (M1-M5 and M7) tested in this study. The alignments are shown for *Tauraco erythrolophus*. Dots indicate residues identical in both copies. The sequences were aligned with MUSCLE [37] in MEGA [36]; Figure S2: Patterns of the PCR products representing M6 marker obtained for male (M) and female (F) individuals of fourteen turaco species (S1–S14, see Table 1). The amplicons were resolved in 2% agarose gel. Lane 1 kb-GeneRuler 1 kb DNA Ladder (Thermo Scientific). Lane LR-FastRuler Low Range DNA Ladder (Thermo Scientific).
