# Supplementary material for: In Silico Analysis of Seven PCR Markers Developed from the CHD1, NIPBL and SPIN Genes Followed by Laboratory Testing Shows How to Reliably Determine the Sex of Musophagiformes Species
Source: Genes (Basel). 2022 May 23;13(5):932. doi: 10.3390/genes13050932 (PMC9140868; doi:10.3390/genes13050932)
Supplement: Supplementary file 1 [file genes-13-00932-s001.zip › Figure S1.pdf]

A)

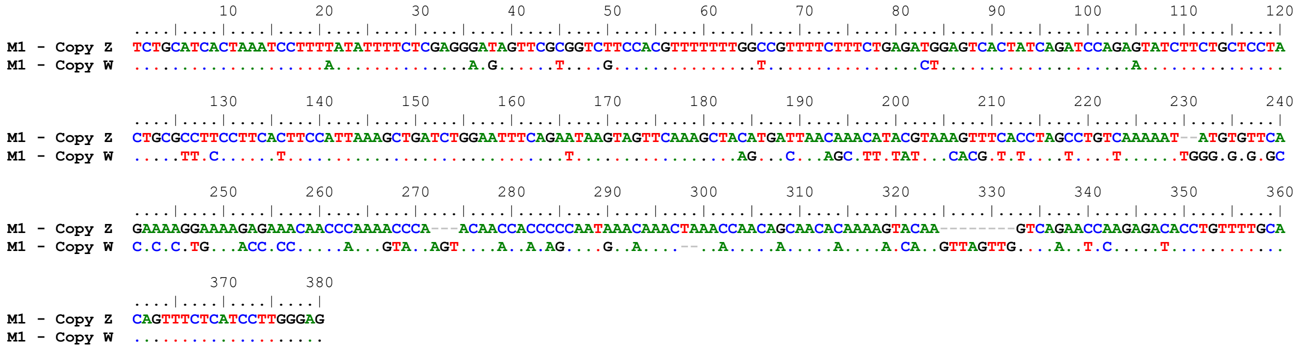

B)

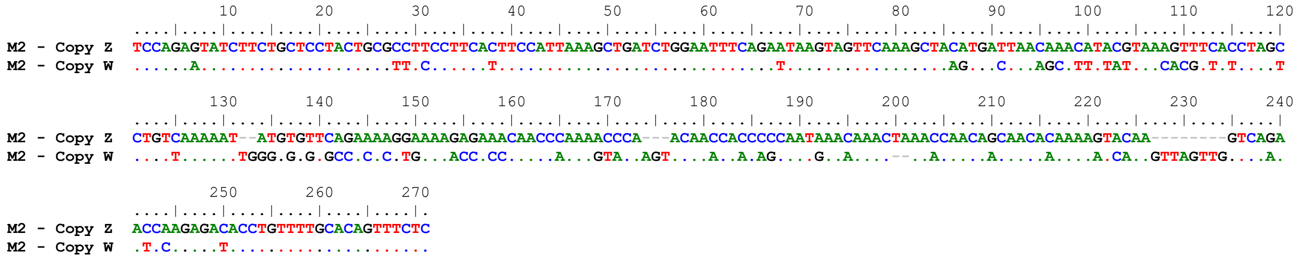

C)

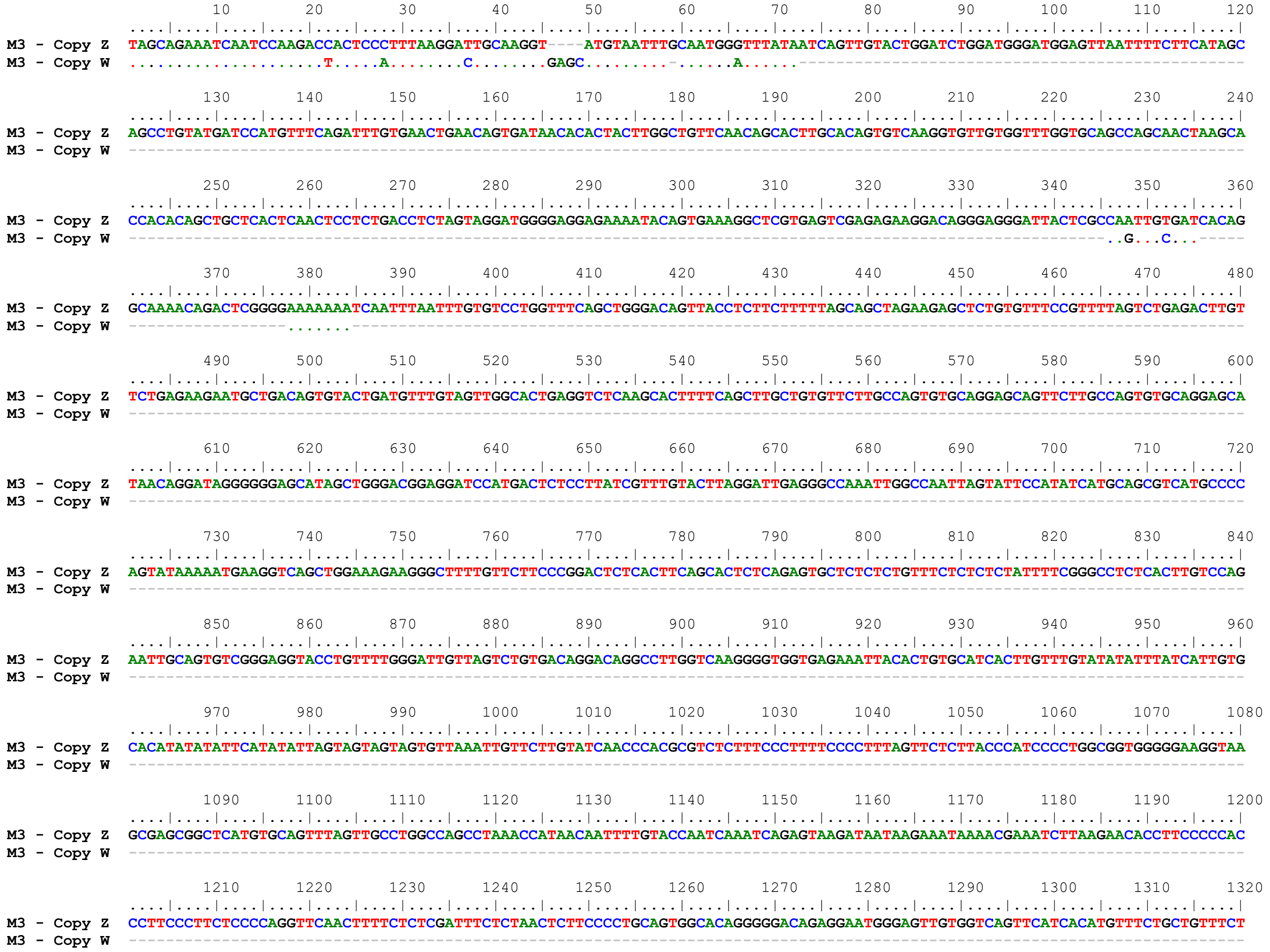

```
                    1330      1340      1350      1360      1370      1380      1390      1400      1410      1420      1430      1440
              ....|....|....|....|....|....|....|....|....|....|....|....|....|....|....|....|....|....|....|....|....|....|....|....|
M3 - Copy Z   TCCTCCTCAGGGGCACTCTTCCTCACACTCTTGCCCTGCTCCAGCATGGGATCCCTCCCATTGGAGATAGTCCTCCATGAACTTACCTGACATGAATCCTTCCCACAGGCTGCAGTTCTT
M3 - Copy W   ------------------------------------------------------------------------------------------------------------------------

                    1450      1460      1470      1480      1490      1500      1510      1520      1530      1540      1550      1560
              ....|....|....|....|....|....|....|....|....|....|....|....|....|....|....|....|....|....|....|....|....|....|....|....|
M3 - Copy Z   CACGAACTGCTGCAGTGTGGGTCCCACCCATGGGGTGCAGTCCTTCACCAAAAGACTGCTCCAGTGCAGGTTTCCCACAGAGTCACGGTCACCTTCAGGCACAGCCATCTACTCCAGCAT
M3 - Copy W   -------------------------------------------------.......----------------------------------------------------------------

                    1570      1580      1590      1600      1610      1620      1630      1640      1650      1660      1670      1680
              ....|....|....|....|....|....|....|....|....|....|....|....|....|....|....|....|....|....|....|....|....|....|....|....|
M3 - Copy Z   GGGATTGTCCACAGGCTGCAGGTGGATCTCTACTCCACTGTTAACCTCCATGGGCTGCAAGGGGACATCTTGTCTCACCACAGGCTGCAGGGGAATCTCAGCTCCAGCACTCCTCCTCCC
M3 - Copy W   ------------------------------------------------------------------------------------------------------------------------

                    1690      1700      1710      1720      1730      1740      1750      1760      1770      1780      1790      1800
              ....|....|....|....|....|....|....|....|....|....|....|....|....|....|....|....|....|....|....|....|....|....|....|....|
M3 - Copy Z   TCTCCTTCCCTGACTTTAGTGTCTTCGTAGTTGTTTCTCCAACATCCCACTCCTCTCTCCTCATTGCTGTGCCAGGCCTGACCAGCCTGACGAAGCTTGGTGGCCCCAGTCACAGACAGC
M3 - Copy W   ------------------------------------------------------------------------------------------------------------------------

                    1810      1820      1830      1840      1850      1860      1870      1880      1890      1900      1910      1920
              ....|....|....|....|....|....|....|....|....|....|....|....|....|....|....|....|....|....|....|....|....|....|....|....|
M3 - Copy Z   TGCCTACATGGGCACCTGGGGTGATGCCTTTGCCTCTCCACTGCAGGTTTTGCCAGCAGTGGGTTTTTTTTTTCTTCCCTTAAATGTATCACATCACACAGGTGATACCACTGTTGCTG
M3 - Copy W   ------------------------------------------------------------------------------------------------------------------------

                    1930      1940      1950      1960      1970      1980      1990      2000      2010      2020      2030      2040
              ....|....|....|....|....|....|....|....|....|....|....|....|....|....|....|....|....|....|....|....|....|....|....|....|
M3 - Copy Z   ATTTGCTCAGCCTTGGCCAGAGCTGAGTTAGACTTGGAGCCAGGGAAGCTTCTAGCAGCTTCTCATAGGAGCCACCCCTGTAACCCCTCCCTCACTACCAAAACACTACTACACAAACCC
M3 - Copy W   ------------------------------------------------------------------------------------------------------------------------

                    2050      2060      2070      2080      2090      2100      2110      2120      2130      2140      2150      2160
              ....|....|....|....|....|....|....|....|....|....|....|....|....|....|....|....|....|....|....|....|....|....|....|....|
M3 - Copy Z   AATACTCAGGGCTGCCTTTGTTTCTTGCTGTGCTGTGCCCACCATGAGTAGACTGGGACTTGGCAAAGGGCTGGGAGGGAACACAGCCAGTATAGCTGACCACAAGTCACCAAAGAAGTA
M3 - Copy W   ------------------------------------------------------------------------------------------------------------------------

                    2170      2180      2190      2200      2210      2220      2230      2240      2250      2260      2270      2280
              ....|....|....|....|....|....|....|....|....|....|....|....|....|....|....|....|....|....|....|....|....|....|....|....|
M3 - Copy Z   TTTCGTGCTATGTAATGTCATGCTCAGCAATAAAACTGGGTGGGCTGAGCAGGGAGTATAAAGGTGGCTGTTGCTTGGAGACTTGCTGGTGAGTTAATATGTGACTCTCTTTCAGTCACT
M3 - Copy W   ------------------------------------------------------------------------------------------------------------------------

                    2290      2300      2310      2320      2330      2340      2350      2360      2370      2380      2390      2400
              ....|....|....|....|....|....|....|....|....|....|....|....|....|....|....|....|....|....|....|....|....|....|....|....|
M3 - Copy Z   TGTGGGTTTTTTTTTGGTTGTAGGTTTTGCTTTGGTTGTTTTTTTCCCTTCACCCAGTGAATTCTTTATTTTGACCCACAAGTTTTCCTCACTTTCACCCTTTCAGTTCTCTCCCTTGTC
M3 - Copy W   ------------------------------------------------------------------------------------------------------------------------

                    2410      2420      2430      2440      2450      2460      2470      2480      2490      2500      2510      2520
              ....|....|....|....|....|....|....|....|....|....|....|....|....|....|....|....|....|....|....|....|....|....|....|....|
M3 - Copy Z   CTGCTAGTGTGGTAGTGAGTAAGGAACTCAGTGGCAGCTTAGCGGCTGGCCAGGTTCAAACCACCACATCAGTTGTGATATTAAAAATCTTGTATGAATCCAGAGATAAGGTAGATATAA
M3 - Copy W   --------------------------------------------------------------------------------------..A..T....C.......A.....A..C..G.T.

                    2530      2540      2550      2560      2570      2580      2590      2600      2610      2620      2630      2640
              ....|....|....|....|....|....|....|....|....|....|....|....|....|....|....|....|....|....|....|....|....|....|....|....|
M3 - Copy Z   TGAAATATTTTTGAAGGTTTTTCAGAATAATAATAATGATA--TATAGTAATATATATGGAAAATATCACTACTAAG--TAACTTTTTGTAGATACCAGTCCCCTGTGT-TTTTACTGAT
M3 - Copy W   .A.............T...G.C.....C.G...........GC...CT.T.G....G.A.C..G........T....AAC.G.C...........TTT..T....ACT.A.....T.ACC

                    2650      2660      2670      2680      2690      2700      2710      2720      2730      2740      2750      2760
              ....|....|....|....|....|....|....|....|....|....|....|....|....|....|....|....|....|....|....|....|....|....|....|....|
M3 - Copy Z   GTTCAAAGATAGGCTTGCTTAGTTTCGTAGTTTATTTTAAAAGTAAACTT--TGAGTGGGTTAATATCACTCCCATTGTGAAATTTAGAGTAGCAATCAAACTAATTTCAGCTTGCAAAT
M3 - Copy W   A.........G.AA...T.....ACTT..A...G............C...TG...A..A...C..G.....TTTG.....G........TC....G............T...........

                    2770      2780      2790      2800      2810      2820      2830      2840      2850      2860      2870      2880
              ....|....|....|....|....|....|....|....|....|....|....|....|....|....|....|....|....|....|....|....|....|....|....|....|
M3 - Copy Z   TGCTTGATCTTT-----TGGCCTAGTTTTCATCAATGGATAAAGACTAGACTTGGTGACCCAGTAGATCCCTACT--TGCTTTTTTTTCTAGAGGAAGACATTTTCGTAACTTCAGTCAG
M3 - Copy W   G.G.CA..A..GTTAAA..........A..TGTG...A......................A.AC....TTG....GC...-...G......T.A..CA.TG....G....T....C.T..

                    2890      2900      2910      2920      2930      2940      2950      2960      2970      2980      2990      3000
              ....|....|....|....|....|....|....|....|....|....|....|....|....|....|....|....|....|....|....|....|....|....|....|....|
M3 - Copy Z   TAGTAGGACTGATGAGTGATTTGCTTTTGACAGGTTCTAAAACAGAGGCCAAGGTTTGTTGCACTAAAGAAGCAACCATCTTATATTGGAGGACACGAAAGTTTAGAGTTAAGAGATTAT
M3 - Copy W   ..............CTCA...............AG............................................................T..G.....................

                    3010
              ....|....|....|
M3 - Copy Z   CAGTTAAATGGACTG
M3 - Copy W   ...............
```

D)

```
                      10        20        30        40        50        60        70        80        90       100       110       120
              ....|....|....|....|....|....|....|....|....|....|....|....|....|....|....|....|....|....|....|....|....|....|....|....|
M4 - Copy Z   GCTACTGATTCGTCTGCGAGAACGTGGCAACAGAGTCCTAATTTTCTCACAGATGGTGAGGATGCTGGACATCCTAGCAGAATATCTGAAGTATCGTCAGTTTCCCTTTCAGGTAAGAAT
M4 - Copy W   ...............A....................A..G........T...........A.....A..................T...................T..............

                     130       140       150       160       170       180       190       200       210       220       230       240
              ....|....|....|....|....|....|....|....|....|....|....|....|....|....|....|....|....|....|....|....|....|....|....|....|
M4 - Copy Z   CTTGGTGGTAGTAGCCAAGAAACTTTGATCTTGAAAATAAGGAAAGTCGTTTTCTTATAACCTCAACTGTTTTGTGTTTATGTGATCTTTACTGCTTTGCTTAAGAAAAGATACAAGAAA
M4 - Copy W   T...C..........A.....G.C...G....T.------------C.AC...A.CT...---...-------------.A...TC....----------..GTAG......TT.T....

                     250       260       270       280       290       300       310       320       330       340       350       360
              ....|....|....|....|....|....|....|....|....|....|....|....|....|....|....|....|....|....|....|....|....|....|....|....|
M4 - Copy Z   AAGTGTTCTTTTTTTCTAG-AAAGACTGGCAATTGCTATGTACCTAATAGTATTTTG-AATTAAACTGATAAATTAAAAACTATGTAAAGTGTTGCATTAATTTTTTATTTCCTTCACAT
M4 - Copy W   GTT.AA.T..A.G.A.AG.A........A.G...A....A.G.TA......G.....A...G.....C..G.....G..---.GA.G.......A.....--C.C.C...C.....C.CC

                     370       380       390       400       410       420       430       440       450       460       470       480
              ....|....|....|....|....|....|....|....|....|....|....|....|....|....|....|....|....|....|....|....|....|....|....|....|
M4 - Copy Z   -AACAGTTTTGGCAGTTAGGAATTCAAGTAGCTCTGATTTTGAATATAGTATAAGAATTACTTTTTAACTGTAGTTTTCAATCTCTTTAGAGACTTGATGGATCAATAAAAGGGGAATTG
M4 - Copy W   A..TT......A..A..GA..........T.......-..A........--..G..G..C.............T.A..A.....................................C...
```

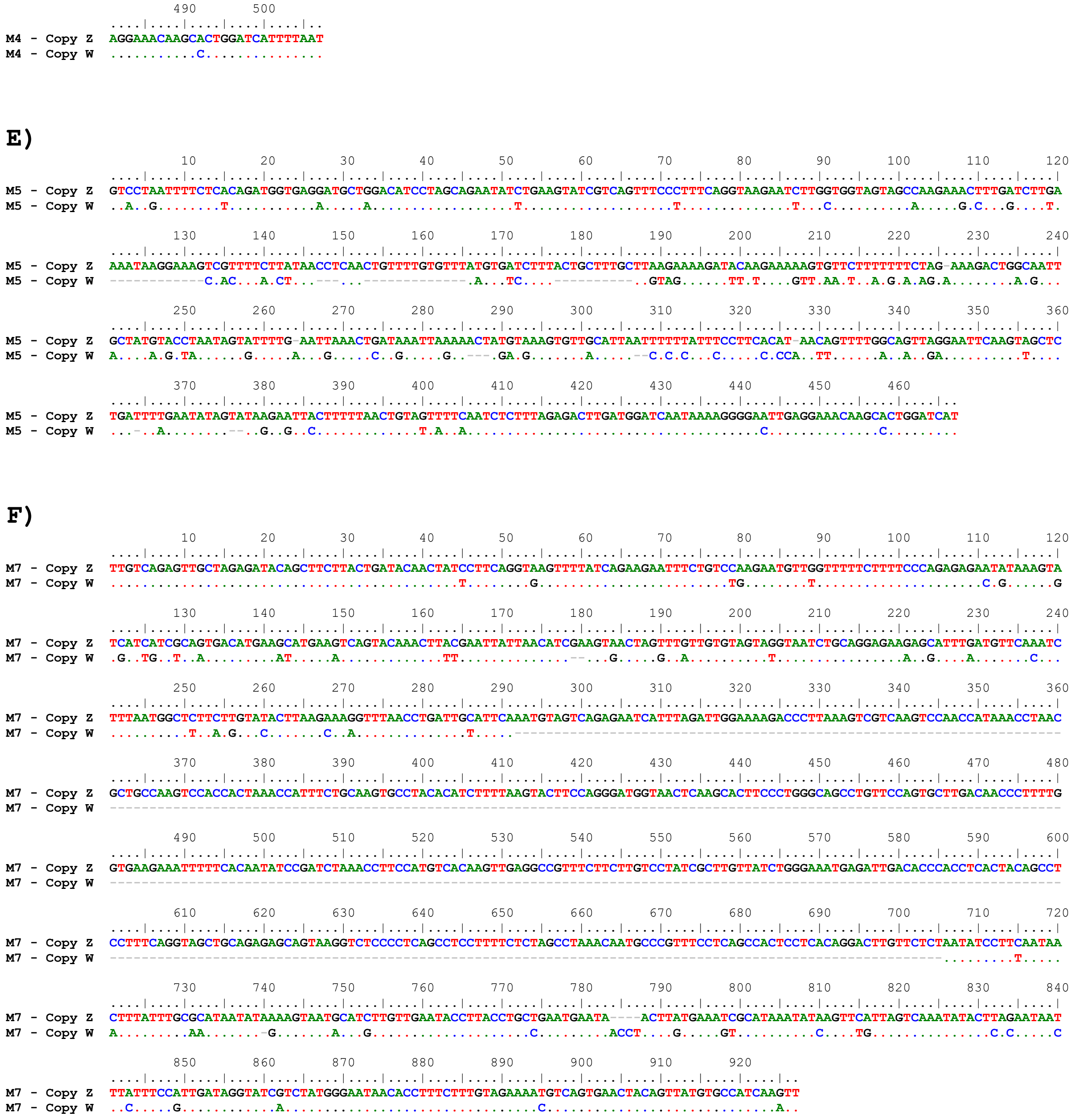

Figure S1. Two-copy (Z and X-specific) sequence comparison of six markers (M1-M5 and M7) tested in this study. The alignments are shown for *Tauraco erythrolophus*. Dots indicate residues identical in both copies. The sequences were aligned with MUSCLE [37] in MEGA [36].
