# Supplementary material for: In Silico Analysis of Seven PCR Markers Developed from the CHD1, NIPBL and SPIN Genes Followed by Laboratory Testing Shows How to Reliably Determine the Sex of Musophagiformes Species
Source: Genes (Basel). 2022 May 23;13(5):932. doi: 10.3390/genes13050932 (PMC9140868; doi:10.3390/genes13050932)
Supplement: Supplementary file 1 [file genes-13-00932-s001.zip › Figure S2.pdf]

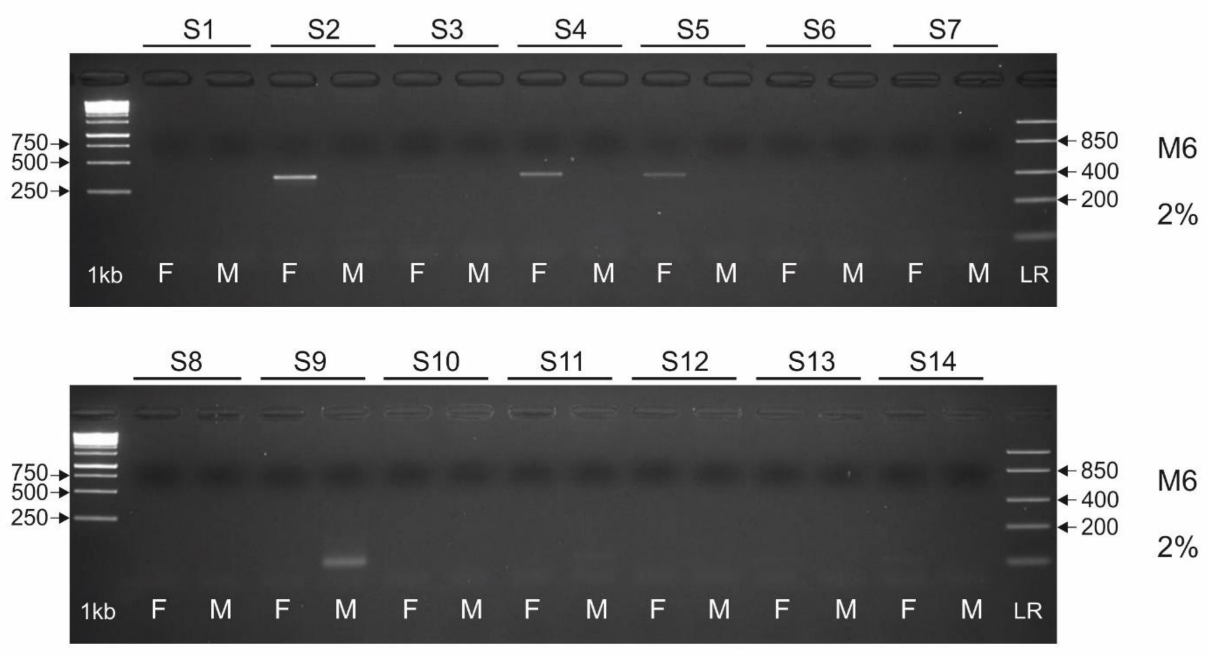

Figure S2. Patterns of the PCR products representing M6 marker obtained for male (M) and female (F) individuals of fourteen turaco species (S1 – S14, see Table 1). The amplicons were resolved in 2% agarose gel. Lane 1kb - GeneRuler 1 kb DNA Ladder (Thermo Scientific). Lane LR - FastRuler Low Range DNA Ladder (Thermo Scientific).
